# Autophagy–Lysosomal Dysfunction as a Converging Mechanism of Cardiomyopathy in Lysosomal Storage Disorders: From Pathobiology to Targeted Therapy

**DOI:** 10.3390/ijms27146418

**Published:** 2026-07-19

**Authors:** Chung-Lin Lee, Chih-Kuang Chuang, Ya-Hui Chang, Huei-Ching Chiu, Yuan-Rong Tu, Yun-Ting Lo, Jun-Yi Wu, Hsiang-Yu Lin, Shuan-Pei Lin

**Affiliations:** 1 Department of Pediatrics, MacKay Memorial Hospital, No. 92, Sec. 2, Zhongshan N. Rd., Taipei 104217, Taiwan; clampcage@gmail.com (C.-L.L.);; 2 Institute of Clinical Medicine, National Yang-Ming Chiao-Tung University, Taipei 112304, Taiwan; 3 International Rare Disease Centre, MacKay Memorial Hospital, Taipei 104217, Taiwan; 4 Department of Medicine, Mackay Medical College, New Taipei City 252005, Taiwan; 5 Department of Nursing, Mackay Junior College of Medicine, Nursing and Management, New Taipei City 252005, Taiwan; 6 Division of Genetics and Metabolism, Department of Medical Research, MacKay Memorial Hospital, Taipei 104217, Taiwan; 7 College of Medicine, Fu-Jen Catholic University, New Taipei City 242062, Taiwan; 8 Department of Medical Research, China Medical University Hospital, Taichung 404327, Taiwan; 9 Department of Infant and Child Care, National Taipei University of Nursing and Health Sciences, Taipei 112303, Taiwan

**Keywords:** lysosomal storage disorders, cardiomyopathy, autophagy–lysosomal dysfunction, Danon disease, TFEB, gene therapy, mucopolysaccharidosis

## Abstract

Cardiac disease is a leading cause of morbidity and early death across several lysosomal storage disorders (LSDs); however, the cardiomyopathies of Fabry, Pompe, and Danon disease are still largely treated as separate, substrate-specific disorders. We argue that they are better understood as variations on a single theme: the breakdown of the autophagy–lysosome system within cardiomyocytes. In the healthy heart, this system clears damaged proteins and organelles and is regulated by mTORC1 and the master regulator TFEB. Once lysosomal degradation or autophagosome–lysosome fusion fails, undegraded substrates and defective mitochondria accumulate, driving hypertrophy, interstitial fibrosis, and conduction disease. Danon disease, resulting from the loss of LAMP2, is the clearest example of a primary defect in autophagic flux, whereas the glycogen storage of Pompe disease and the globotriaosylceramide accumulation of Fabry disease impair flux through different upstream mechanisms that converge on the same downstream injury. The same framework extends to other storage disorders with cardiac involvement, such as mucopolysaccharidosis (MPS). We trace this shared pathobiology from molecule to bedside, examine biomarkers that reflect lysosomal and autophagic dysfunction rather than storage alone, and re-examine treatment in that light: why enzyme replacement therapy corrects substrate accumulation but leaves much of the autophagic and mitochondrial damage unresolved, and why gene therapy—particularly AAV9-LAMP2B for Danon disease—together with autophagy- and TFEB-directed strategies may help close that gap. Viewing these disorders through a single mechanistic lens reshapes how we monitor them and where future therapies should be directed.

## 1. Introduction

The lysosomal storage disorders (LSDs) are a family of inherited metabolic diseases in which a defect in a single lysosomal enzyme, transporter, or membrane protein impairs the turnover of a specific class of macromolecules. The undegraded substrate accumulates, and although the genetic lesion is present in every cell, the resulting clinical damage is concentrated in tissues least able to tolerate the burden. The heart is consistently one such tissue. In Fabry, Pompe, and Danon disease—three otherwise distinct LSDs—cardiac involvement is not a late manifestation but a major determinant of morbidity and survival [1,2,3]. Fabry cardiomyopathy alone accounts for a notable proportion of unexplained left ventricular hypertrophy: approximately 1% of patients with a clinical diagnosis of hypertrophic cardiomyopathy (HCM) have Fabry disease [2]. Because the cardiac phenotype often develops without the systemic “red flags” of the classic syndrome, these patients are frequently diagnosed late, after hypertrophy, fibrosis, and conduction abnormalities have already become established [2,4,5].

Historically, each of these disorders has been understood—and treated—according to the substrate that accumulates. Fabry disease is viewed as a consequence of globotriaosylceramide (Gb3) accumulation resulting from α-galactosidase A deficiency [1,4]; Pompe disease as lysosomal glycogen accumulation resulting from acid α-glucosidase (GAA) deficiency [6]; and Danon disease as a vacuolar cardiomyopathy caused by the loss of lysosome-associated membrane protein-2 (LAMP-2) [3]. Therapeutic strategies have largely followed the same logic, most notably in enzyme replacement therapy, in which a recombinant enzyme is administered to clear a specific substrate. Although this substrate-centered view has been productive, its limitations are increasingly apparent. It is important to be clear that these remain three genetically distinct diseases, each with its own primary defect, and correcting that defect is and should be the first aim of treatment. The limitation is conceptual rather than a statement about any individual patient: by attending only to the substrate that accumulates, this view treats mechanistically related disorders in isolation, gives little insight into why clearing the primary substrate—whether by enzyme replacement or by other means—can improve circulating and storage markers yet frequently fails to halt myocardial deterioration, and obliges the cardiac phenotype of each disease to be explained on its own terms [4,6,7].

An alternative perspective is possible. What Fabry, Pompe, and Danon disease share is not a substrate but a common pathway: the autophagy–lysosome system through which cardiomyocytes degrade and recycle proteins and organelles. Danon disease provides the clearest example, as loss of LAMP-2 directly impairs autophagosome–lysosome fusion, leading to the accumulation of autophagic vacuoles as a primary event [3,8,9]. In Pompe disease, lysosomal rupture and glycogen overload disrupt autophagic flux, resulting in the accumulation of undegraded autophagic debris in striated muscle [6,7]. In Fabry disease, Gb3 storage is accompanied by lysosomal dysfunction and, increasingly recognized, by mitochondrial and inflammatory injury that extends well beyond simple substrate deposition [1,4]. Across all three disorders, the downstream consequences converge: impaired clearance of damaged mitochondria, energetic and oxidative stress, hypertrophic and profibrotic signaling, and electrical remodeling. This pattern has been documented even at the level of mitochondrial fragmentation in Danon disease [8]. Viewed in this way, the three “substrate diseases” represent distinct entry points into a common failure of cardiac proteostasis.

**Figure 1:**
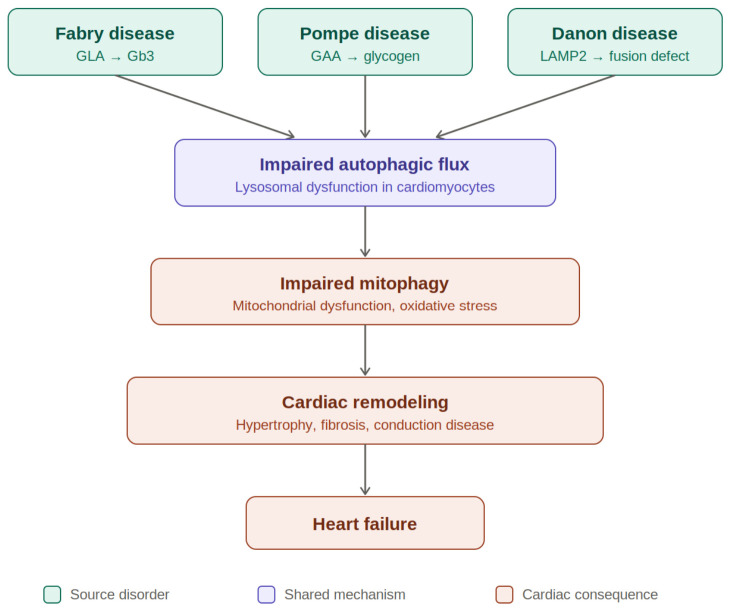
Distinct upstream lesions in Fabry, Pompe, and Danon disease converge on impaired autophagic flux and lysosomal dysfunction in cardiomyocytes, driving impaired mitophagy, cardiac remodeling, and ultimately heart failure.

This reframing is timely for a practical reason. Treatment is moving beyond substrate clearance toward correction of the underlying genetic and cellular defects. Oral pharmacological chaperones and adeno-associated virus (AAV)-based gene therapies are currently being evaluated in early-phase clinical trials for Pompe and Danon disease, including AAV-mediated delivery of LAMP2B [3,6,7,8]. The success of these approaches depends on their ability to restore autophagic and lysosomal function, rather than simply reduce storage burden. As a result, a mechanism-based understanding that spans multiple diseases is directly relevant to diagnosis, monitoring, and treatment. In this review, we propose a common framework for understanding these disorders. We first outline the autophagy–lysosome system in cardiomyocytes and then examine Fabry, Pompe, and Danon disease as variations of a shared defect (summarized in Table 1, with the convergent mechanism illustrated in Figure 1). We also consider biomarkers and imaging modalities that reflect autophagic dysfunction rather than storage status alone and reassess current and emerging therapies through the same mechanistic lens.

## 2. Autophagy–Lysosome System in the Cardiomyocyte

The cardiomyocyte is a long-lived, postmitotic cell that contracts continuously and cannot dilute damaged components through cell division. Therefore, it depends more than most cells on continuous quality-control mechanisms—including the ubiquitin–proteasome system, molecular chaperones, and autophagy—to maintain proteome and organelle integrity [10]. Of these, macroautophagy (hereafter referred to as autophagy) is the pathway responsible for clearing material that the proteasome cannot handle, including protein aggregates and whole organelles. The quantity that matters is not how many autophagosomes are present at a given moment but rather autophagic flux—the throughput of the entire process, from phagophore nucleation and cargo capture through autophagosome formation, fusion with lysosomes, and degradation of the contents by acid hydrolases, with the breakdown products returned to the cell [10,11]. This distinction is not merely academic: when a late step, such as autophagosome–lysosome fusion or lysosomal degradation, fails, autophagosomes and undegraded cargo accumulate even as effective clearance collapses, resulting in high vacuole counts despite low flux.

Because the heart’s contractile activity imposes an unrelenting demand for ATP, the quality of its mitochondrial pool is under constant scrutiny. Damaged mitochondria—which generate reactive oxygen species and release proapoptotic factors—must therefore be cleared promptly by mitophagy, the selective autophagy of mitochondria [11]. Two pathways converge on this process: the ubiquitin-dependent PINK1/Parkin pathway, in which PINK1 accumulates on depolarized mitochondria and Parkin ubiquitinates outer membrane proteins to recruit autophagy receptors, and receptor-mediated mitophagy through BNIP3, NIX, and FUNDC1 [11]. Importantly, mitophagy feeds into the same autophagosome–lysosome machinery used for bulk turnover. As a result, defects in lysosomal function or autophagosome–lysosome fusion impair protein clearance and mitochondrial clearance, helping to explain why a primarily lysosomal defect ultimately leads to mitochondrial dysfunction in the myocardium [11,12].

Autophagic and lysosomal capacity are not fixed but are coordinated transcriptionally by the MiT/TFE family of transcription factors, particularly TFEB and TFE3, which drive the coordinated lysosomal expression and regulation (CLEAR) gene network governing lysosomal biogenesis, autophagy-related genes, and lipid catabolism [13]. Their activity is regulated largely by nutrient and stress signaling. Under nutrient-replete conditions, mTORC1 phosphorylates TFEB and retains it in the cytosol, whereas starvation, energetic stress (acting in part through AMPK), or lysosomal stress relieves this restraint, allowing TFEB to translocate to the nucleus and expand autophagic and degradative capacity [13]. In principle, this provides the cardiomyocyte with a demand-matched reserve—the ability to scale lysosomal capacity as cellular stress increases—a reserve that becomes increasingly important and is ultimately overwhelmed when an inherited defect floods the lysosome with undegradable substrate.

**Figure 2:**
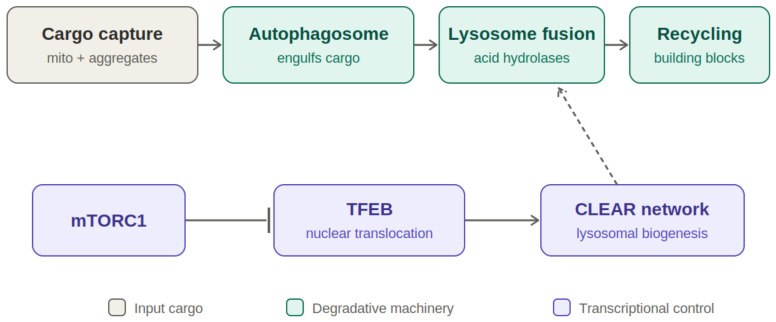
Autophagy–lysosome system in the cardiomyocyte. Protein aggregates and damaged mitochondria are captured within autophagosomes, which fuse with lysosomes for degradation by acid hydrolases and recycling of the resulting breakdown products. Mitophagy (PINK1/Parkin- and receptor-mediated) delivers cargo into the same pathway. The MiT/TFE transcription factor TFEB, retained in an inactive state by mTORC1, translocates to the nucleus under stress to activate the CLEAR network and expand lysosomal capacity (dashed arrow).

Three features make the heart particularly vulnerable when this system falters. Cardiomyocytes are postmitotic; therefore, damage that other tissues might dilute through cell division instead accumulates; they contain a dense, high-output mitochondrial population whose turnover depends on intact mitophagy; and their contractile and electrical functions rely on a precisely maintained sarcomeric and organellar architecture that autophagy continuously prunes [10,12]. When autophagic flux declines, the consequences—retained substrate, accumulation of dysfunctional mitochondria, oxidative stress, and disturbed calcium handling—directly affect contraction and conduction [12]. This vulnerability is precisely what lysosomal storage cardiomyopathies exploit, and Danon disease illustrates it in its purest form: loss of the fusion-enabling protein LAMP-2 blocks autophagosome–lysosome fusion, autophagic vacuoles accumulate, and the myocardium hypertrophies and ultimately fails [3]. The sections that follow examine how Fabry, Pompe, and Danon disease each enter this shared pathway through a different mechanism. Figure 2 summarizes the machinery on which they converge.

## 3. One Mechanism, Three Diseases

### 3.1. Danon Disease (LAMP-2): A Primary Block in Autophagosome–Lysosome Fusion

LAMP-2 is a lysosomal membrane protein whose LAMP-2B isoform is required for autophagosome–lysosome fusion. Therefore, loss-of-function variants in the X-linked LAMP2 gene arrest autophagy at the fusion step, resulting in the accumulation of undegraded autophagic vacuoles with sarcolemmal features in cardiomyocytes—the histological hallmark of the disease [3]. Clinically, this produces severe, often early-onset HCM: males typically exhibit a triad of cardiomyopathy, myopathy, and intellectual disability, whereas females more often present with a cardiomyopathy-predominant phenotype, and heart failure is the leading cause of death [3,9]. Recent work has extended this picture beyond a purely lysosomal defect, implicating LAMP-2 deficiency in mitochondrial fragmentation and dysfunction as a driver of disease progression rather than a bystander effect [8]. Danon disease is thus the clearest demonstration of the argument advanced here: disruption of the fusion step alone is sufficient to produce a storage-type cardiomyopathy. It is also the disorder in which disease-modifying therapy is most advanced, with AAV-mediated LAMP2B delivery currently undergoing clinical evaluation [3,8].

### 3.2. Pompe Disease (GAA): Lysosomal Glycogen Overload That Impairs Autophagic Flux

One step upstream, Pompe disease begins not with a fusion defect but with a substrate that the lysosome cannot clear. Deficiency of acid α-glucosidase (GAA), inherited in an autosomal recessive manner, blocks lysosomal glycogen breakdown, causing glycogen-engorged lysosomes to enlarge and rupture in cardiac and skeletal muscle [6]. The infantile-onset form, characterized by near-absent enzyme activity, presents with severe HCM during the initial months of life, whereas late-onset disease is dominated by skeletal muscle and respiratory involvement [6]. However, the lesion is not simply one of substrate storage. As glycogen accumulates, autophagic flux becomes disrupted and undegraded autophagic debris accumulates within myofibers, aggravating muscle injury and impairing the delivery of replacement enzymes. This may help explain why enzyme replacement therapy rescues the infantile heart more effectively than skeletal muscle [6,7]. Nevertheless, enzyme replacement therapies (alglucosidase alfa and avalglucosidase alfa) have transformed survival in infantile-onset Pompe disease, and AAV-based gene therapy has entered phase I/II clinical testing [6,7].

### 3.3. Fabry Disease: Substrate Storage with Secondary Autophagic and Mitochondrial Injury

Fabry disease lies at the indirect end of the spectrum. Deficiency of α-galactosidase A, encoded by the X-linked GLA gene, leads to the accumulation of globotriaosylceramide (Gb3) and its deacylated form, lyso-Gb3, in cardiomyocytes, the conduction system, and the vasculature, resulting in concentric left ventricular hypertrophy, myocardial fibrosis, diastolic dysfunction, and arrhythmias. Cardiac disease remains the leading cause of death in affected patients [1]. Here, the primary lesion is glycosphingolipid storage rather than a direct defect in the autophagy machinery. However, substrate storage alone does not fully explain the disease process. Impaired autophagic flux, lysosomal dysfunction, mitochondrial abnormalities, and chronic inflammation are increasingly recognized components of Fabry cardiomyopathy and may help explain why cardiac disease can progress despite substrate-directed therapy [1,4]. Because as many as 1% of patients diagnosed with HCM have Fabry disease and the cardiac phenotype is often recognized late, multimodality imaging—particularly T1 mapping—and measurement of lyso-Gb3 have become central to early diagnosis and disease monitoring [2,5]. Current treatment includes enzyme replacement therapy and the oral pharmacological chaperone migalastat for patients with amenable variants, whereas gene therapy and substrate reduction approaches remain under development [2,4].

### 3.4. Beyond the Core Three

The same framework extends to other storage and glycogen-handling disorders with cardiac involvement. In mucopolysaccharidoses, accumulation of glycosaminoglycans within the myocardium and cardiac valves produces left ventricular hypertrophy and progressive valvular thickening. Although enzyme replacement therapy improves some myocardial parameters, it has limited effects on established valvular disease, illustrating a tissue-specific limitation of substrate clearance that echoes the therapeutic gap observed in Fabry and Pompe disease [14]. PRKAG2 syndrome, caused by mutations in the γ2 regulatory subunit of AMP-activated protein kinase, is a glycogen-storage cardiomyopathy that mimics sarcomeric HCM while featuring ventricular pre-excitation and conduction disease. It serves as a reminder that abnormal cardiac glycogen handling need not be lysosomal in origin to produce a similar hypertrophic and arrhythmogenic phenotype [15]. Other lysosomal and autophagy-related disorders, including the mucolipidoses, involve the heart less consistently but follow the same general theme: once degradative capacity can no longer keep pace with substrate burden, the myocardium undergoes hypertrophy, fibrosis, and conduction abnormalities, regardless of the nature of the upstream defect.

Inheritance also shapes who develops cardiac disease and when. Two of the three core disorders—Fabry and Danon disease—are X-linked, and their cardiac expression therefore differs between the sexes. In Danon disease, hemizygous males typically present in adolescence with severe, early-onset cardiomyopathy, whereas heterozygous females tend to develop a later and somewhat milder, though still progressive, phenotype [3,9]. Fabry disease follows a comparable pattern but with wider variation: because of random X-chromosome inactivation, heterozygous women range from nearly asymptomatic to fully affected, frequently develop cardiac-predominant disease at older ages, and are more often diagnosed late or missed by enzyme-based screening [16]. Pompe disease, which is autosomal recessive, affects both sexes equally. These differences are not incidental: sex influences the timing of surveillance, the interpretation of biomarkers, and the decision of when to begin treatment.

## 4. Convergent Downstream Injury

Regardless of where a disorder enters the autophagy–lysosome pathway, the early cellular consequence is the same: a backlog of undegraded material and a failure to remove damaged organelles. The mitochondrion is the most consequential casualty. When mitophagy cannot keep pace, damaged mitochondria persist, generate reactive oxygen species, and reduce the threshold for the opening of the permeability transition pore, compounding oxidative and energetic stress and, in severe cases, triggering cardiomyocyte death [11,12]. This is no longer a hypothetical link: in Danon disease, loss of LAMP-2 has been directly associated with mitochondrial fragmentation and dysfunction, demonstrating how a defect nominally confined to the lysosome can propagate to the organelle that the heart can least afford to lose [8].

A heart working under this internal handicap responds as it does to any sustained stress—with hypertrophy. Storage-laden, energy-deprived cardiomyocytes enlarge, and their sarcomeric and organellar architecture becomes distorted by accumulated substrate as well as by the expanded autophagic and lysosomal compartments themselves. At the same time, mitochondrial dysfunction and reactive oxygen species promote the maladaptive signaling pathways that drive this growth [12]. The result, across Fabry, Pompe, and Danon disease alike, is the concentric left ventricular hypertrophy that first brings many of these patients to clinical attention [1].

Sustained injury and cardiomyocyte loss do not resolve cleanly. Dying cells and the associated inflammatory response recruit and activate fibroblasts, which differentiate into matrix-secreting myofibroblasts under the influence of transforming growth factor-β and related mediators, resulting in interstitial and replacement fibrosis [17]. In Fabry disease, this fibrosis has a characteristic distribution and is among the strongest predictors of outcome, which is why its detection by late gadolinium enhancement and T1 mapping has become central to disease staging [1,5]. Fibrosis is not merely a record of prior injury; it actively reshapes the mechanical and electrical properties of the heart.

The electrical consequences represent the final common pathway. Fibrosis disrupts orderly conduction, whereas remodeling of ion channels and gap junctions slows impulse propagation and creates a substrate for re-entrant and triggered arrhythmias [18]. Superimposed on this are disease-specific features—such as the ventricular pre-excitation and progressive conduction disease characteristic of Danon disease and PRKAG2 syndrome [9,15]—so that the clinical endpoints converge as clearly as the molecular mechanisms: hypertrophy, fibrosis, conduction block, arrhythmia, and sudden death. Tracing these shared endpoints back to their common origin highlights the value of a mechanistic perspective, suggesting that monitoring and treatment should focus on the integrity of the autophagy–lysosome–mitochondrial axis rather than on the stored substrate alone.

## 5. Biomarkers Beyond Storage

The biomarkers used routinely in clinical practice primarily measure stored substrate. In Fabry disease, plasma lyso-Gb3 is the workhorse biomarker: it performs well for diagnosis and for newborn or high-risk screening when paired with enzyme activity testing or GLA genotyping, is analytically reliable, and is useful for monitoring individual patients over time [19]. Its limitations are particularly relevant in cardiac disease. It is considerably less sensitive in women and in patients with late-onset, cardiac-predominant variants—the very patients most likely to present to a cardiologist—and although plasma levels correlate moderately with cardiac measures across patient groups, they do not reliably reflect the fibrosis that determines prognosis [19]. The pattern is similar in other disorders: urinary glucose tetrasaccharide (Glc4) in Pompe disease and urinary glycosaminoglycans in mucopolysaccharidoses support diagnosis and reflect overall substrate burden but neither provides a faithful measure of myocardial status [6,14]. By definition, a storage marker measures the input to the pathway rather than the downstream machinery that ultimately fails.

Markers that reflect the state of the heart itself perform better as prognostic tools. High-sensitivity cardiac troponin, which reflects ongoing cardiomyocyte injury, and NT-proBNP, which reflects myocardial wall stress, both increase as cardiomyopathy progresses and capture the consequences of the disease rather than its upstream cause. Although nonspecific, they are clinically informative. What remains conspicuously absent is a validated circulating marker of the process that this review places at its center—autophagic flux and lysosomal capacity. No blood test currently reports directly on whether autophagosome–lysosome turnover is functioning adequately. The closest available surrogates reflect downstream consequences, including indices of mitochondrial dysfunction and, increasingly, inflammatory activity, which in Fabry disease correlates with disease stage and may increase before fibrosis becomes established [20]. This remains less a solved problem than a clearly defined unmet need [11,12].

Several candidate markers are beginning to address this gap, although none has yet been validated for cardiac lysosomal disease. Because autophagosome turnover leaves molecular traces, the autophagy proteins LC3-II and p62/SQSTM1—long used to gauge autophagic flux in tissue and now measurable in blood and in circulating extracellular vesicles—have been proposed as surrogates of pathway activity, with the important caveat that their levels reflect the balance between formation and clearance and must be read dynamically rather than as a single static value. Failed mitophagy offers a second readout: mitochondria that are not cleared release mitochondrial DNA into the circulation, and cell-free mtDNA is elevated in heart failure and tracks with systemic inflammation and congestion, making it a plausible reporter of the mitochondrial arm of the injury [21]. Extracellular vesicles shed by cardiomyocytes provide a third, tissue-proximal source, since their protein and microRNA cargo can carry autophagic and mitophagic signatures from the myocardium into the blood [22]. These approaches remain exploratory, but validating any of them against imaging and outcome data in Fabry, Pompe, and Danon disease is among the more tractable steps toward the pathway-level biomarker the field still lacks [11].

Where circulating biomarkers are limited, cardiac imaging has emerged as the most informative indicator of cardiac status, with cardiac magnetic resonance serving as a noninvasive tissue assay. Native T1 mapping is particularly valuable: sphingolipid accumulation reduces native T1 in Fabry disease—a signature that distinguishes it from the elevated T1 seen in amyloidosis and the reduced T1 associated with iron overload—and this change may precede the development of overt hypertrophy. As fibrosis and edema progress, however, T1 values increase, such that pseudonormalization may mask advancing disease [23]. Late gadolinium enhancement identifies replacement fibrosis, characteristically involving the basal inferolateral wall in Fabry disease, and functions as a noninvasive surrogate for tissue biopsy, whereas extracellular volume quantification provides a measure of diffuse interstitial fibrosis that may not be detected by late gadolinium enhancement [23]. These imaging parameters track disease progression from early substrate accumulation to fibrosis, explaining their central role in disease staging and treatment monitoring [2,5]. The trajectory they describe—from low-T1 storage to fibrosis and structural deterioration—mirrors the pathogenic cascade outlined in the preceding sections [1,17].

**Figure 3:**
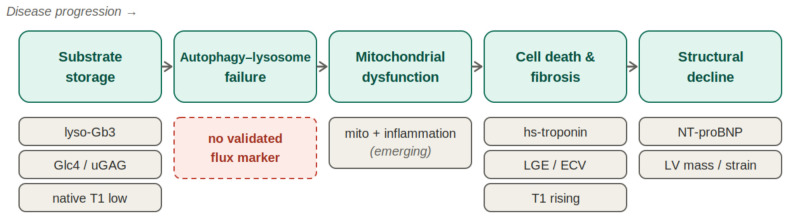
Biomarker classes mapped onto the stages of cardiac injury. Storage markers and native T1 reflect the earliest stage, whereas markers of injury, fibrosis, and wall stress reflect later stages. No validated circulating biomarker currently reports autophagic–lysosomal dysfunction directly (dashed box).

In practice, the most robust assessment of the autophagy–lysosome–mitochondrial axis comes not from any single test but from integrating multiple modalities: a storage marker to establish and monitor the diagnosis, injury and stress markers to assess the cardiac response, and multiparametric imaging to characterize myocardial tissue from early infiltration through fibrosis (summarized in Table 2; Figure 3 maps each biomarker class to the stage of injury it reflects). The unmet need is a biomarker that directly reports the integrity of autophagic and lysosomal function—the very process that current therapies seek to restore but cannot yet directly measure. This gap provides the rationale for the next section: if substrate clearance and pathway function can diverge, treatment should ultimately be judged by the latter and the biomarkers used should be capable of capturing it.

## 6. Rethinking Therapy Through the Same Lens

### 6.1. Reach and Limits of Enzyme Replacement

Enzyme replacement therapy was the first intervention to alter the natural history of several of these diseases. Recombinant acid α-glucosidase (alglucosidase alfa and avalglucosidase alfa) transformed survival in infantile Pompe disease [6,7], and three agents for Fabry disease—agalsidase alfa, agalsidase beta, and pegunigalsidase alfa—have stabilized renal, cardiac, and neurological outcomes [24]. Its limitations, however, are mechanistic. The infused enzyme must reach the lysosome of every affected cell through mannose-6-phosphate-mediated uptake, yet uptake into cardiomyocytes and skeletal muscle is inefficient. Once the autophagic–lysosomal compartment becomes overloaded and fibrosis is established, the delivered enzyme cannot reverse these changes. This helps explain why treatment benefits depend so strongly on early initiation, before irreversible organ fibrosis develops; patients with advanced end-organ damage respond far less well [17,25]. When the burden of lifelong fortnightly infusions and the potential development of neutralizing antibodies that reduce efficacy are also considered [24], enzyme replacement emerges as a therapy directed primarily at the upstream enzyme deficiency rather than the downstream pathway failure that may, by the time cardiac disease becomes clinically apparent, have acquired its own momentum. Mechanistically, replacing the deficient enzyme restores hydrolysis within the lysosomal lumen but does nothing to rebuild the downstream machinery that has already failed—the autophagosome–lysosome fusion apparatus, the complement of functional lysosomes, and the mitophagy that removes damaged mitochondria. By the time cardiac disease is clinically apparent, chronic suppression of autophagic flux has allowed dysfunctional mitochondria, reactive oxygen species, and profibrotic signalling to become self-sustaining, so that lowering the upstream substrate no longer switches off the injury it once initiated [11,12].

### 6.2. Stabilizing the Enzyme and Lowering the Substrate

Two approaches refine the same upstream strategy without requiring protein infusion. The pharmacological chaperone migalastat is a small molecule that binds to and stabilizes amenable (responsive) missense variants of α-galactosidase A, restoring protein folding, trafficking, and residual enzymatic activity. Administered orally every other day, it stabilizes renal function and provides cardiovascular benefit [24]. Its use, however, is limited by genotype, as only patients with amenable variants are eligible, and real-world studies suggest that the in vivo amenability of some variants may be lower than predicted [25]. Substrate reduction therapy addresses the problem from the opposite direction by reducing synthesis of the offending substrate rather than enhancing its clearance, with glucosylceramide synthase inhibition currently under development for Fabry disease [24]. Both strategies are attractive because they are oral and cell-penetrant. However, like enzyme replacement therapy, they primarily alter substrate balance and leave recovery of the autophagy–lysosome machinery to occur indirectly.

### 6.3. Gene Therapy and the Special Case of Danon Disease

Gene therapy seeks to restore the missing protein at its source. For enzyme-deficiency disorders, AAV-mediated delivery of GAA in Pompe disease and GLA in Fabry disease is currently in clinical development, offering the prospect of sustained endogenous protein production and freedom from regular infusions [7,24]. Danon disease is the conceptually clearest target and the most advanced in development, largely because the defect involves not an enzyme but the structural fusion protein LAMP-2. A phase 1 trial of AAV9-delivered LAMP2B (RP-A501) demonstrated myocardial LAMP2 expression and, among patients with preserved baseline ejection fraction, stabilization or reduction of the left ventricular mass accompanied by decreases in troponin I and NT-proBNP over a follow-up period of 2–4.5 years. Reported adverse events included one case of complement-mediated thrombotic microangiopathy and glucocorticoid-related myopathy, both managed with transient immunomodulatory treatment [26]. The observation that a single infusion of the fusion protein can reopen the blocked pathway in the human heart provides strong support for the mechanistic framework outlined in this review [3,8]. An important remaining question, however, is whether the restoration of the initiating defect is sufficient to fully normalize downstream autophagic flux. Several vector-specific hurdles temper this optimism. Because a large proportion of adults carry pre-existing neutralising antibodies against the AAV9 capsid, many patients are ineligible at screening, and the humoral response provoked by a first dose effectively precludes re-treatment. The capsid and the transgene product can also trigger innate and adaptive immune reactions—the complement-mediated thrombotic microangiopathy observed in the RP-A501 trial is one such example—which is why immunomodulation is built into current protocols. Delivery poses a further constraint, since transducing enough of the large, post-mitotic cardiomyocyte population to correct the phenotype is difficult, and the high vector doses needed for cardiac and skeletal-muscle penetration increase hepatic sequestration and the associated risks of liver toxicity and off-target expression [26,27]. Whether durable, cardiac-directed expression can be achieved at tolerable doses is therefore the central practical question for this strategy.

### 6.4. Targeting the Pathway: Autophagy and TFEB

**Figure 4:**
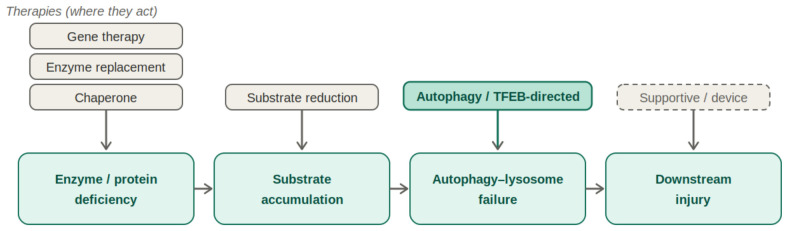
Current and emerging therapies act at different points along the disease pathway. Enzyme replacement, pharmacological chaperones, and gene therapy act at the level of the deficient enzyme or protein; substrate reduction therapy lowers the accumulation of the offending substrate; and only autophagy- and TFEB-directed strategies target the convergent autophagy–lysosome defect itself (highlighted). Supportive and device-based therapies address downstream cardiac injury.

The logical extension of a mechanism-based perspective is to target the autophagy–lysosome axis independently of the underlying genetic defect. TFEB, the master transcriptional regulator of lysosomal biogenesis and autophagy, represents an attractive target. In cardiovascular models, TFEB activation improves cardiac function by alleviating lysosomal and mitochondrial dysfunction while reducing inflammation [13,28]. Approaches under investigation include modulation of mTORC1 and the use of direct TFEB activators to expand degradative capacity and restore autophagic flux. This therapeutic class remains the least mature and carries important caveats. Autophagy is highly context-dependent, and excessive activation may itself be detrimental to the heart. Nevertheless, it is the only strategy aimed directly at the convergent lesion rather than the disease-specific cause and therefore has the potential to complement substrate-directed and gene-directed therapies across these disorders. The central message is straightforward: established therapies act upstream of autophagy–lysosome dysfunction, whereas newer approaches act at or directly upon this pathway. Matching the level of intervention to the level of the lesion—and confirming the effect with biomarkers that reflect pathway function—represents the practical application of viewing these disorders through a common mechanistic framework (Figure 4 and Table 3).

## 7. Open Questions and Future Directions

The framework proposed in this review highlights a specific limitation. We can measure stored substrate and image fibrosis; however, we still cannot directly measure, in a living patient, the variable placed at the center of this discussion—autophagic flux and lysosomal capacity [19]. Until a validated biomarker becomes available, claims that a pathway has been “restored” remain inferential rather than directly measurable, forcing clinical trials to evaluate pathway-directed therapies using downstream surrogate endpoints. Closing this gap represents the highest-priority methodological challenge in the field and would allow the mechanistic framework advanced here to be tested directly rather than inferred indirectly.

Framed in this way, the central claim of this review is falsifiable, and it is worth setting out the predictions that follow from it. The claim is that impaired autophagic flux is the proximate cause of the cardiomyopathy in these disorders rather than an incidental accompaniment of storage. Three predictions follow. First, the degree of flux impairment in cardiomyocytes should track the severity of the cardiac phenotype across Fabry, Pompe, and Danon disease more closely than the quantity of stored substrate does. Second, restoring flux without removing the primary substrate—for instance by activating TFEB—should improve the cardiac phenotype. Third, correcting the primary genetic defect while flux remains blocked should fail to rescue the heart. Each of these can be tested directly.

A tractable experimental programme follows. Patient-derived induced pluripotent stem-cell cardiomyocytes carrying Fabry, Pompe, or Danon mutations, studied side by side, allow autophagic flux to be measured directly with tandem fluorescent LC3 reporters under lysosomal inhibition, alongside assays of mitophagy and respiration, so that flux can be correlated with contractile and hypertrophic readouts within a single system [15]. Epistasis experiments in these lines—suppressing flux by ATG7 or LAMP2 knockdown while enzyme activity is normal, and conversely restoring flux by TFEB or TFE3 gain of function while the primary substrate is left untouched—would test whether flux is both necessary and sufficient for the cellular phenotype. The same logic extends in vivo: delivering a flux-directed intervention such as AAV-mediated TFEB to established disease models and reading out cardiac mass, fibrosis, conduction, and survival would show whether the convergent lesion is a viable target independent of substrate, while comparing gene replacement with and without adjunctive flux enhancement would reveal whether restoring the initiating protein alone normalises downstream autophagy. In patients, finally, relating myocardial autophagic-vacuole burden and imaging measures of tissue remodelling to longitudinal outcomes—and ultimately to a validated circulating marker of flux—would connect the mechanistic model to the clinic. We regard these experiments, rather than further descriptive characterisation of stored substrate, as the priority for the next phase of work.

A second set of questions concerns whom to treat and when. The therapies that perform best are generally those initiated early, before fibrosis becomes irreversible [17], placing a premium on presymptomatic detection. Newborn screening programs for Fabry disease, Pompe disease, and mucopolysaccharidoses now identify affected infants before symptoms appear. However, experience accumulated over more than a decade of population screening has also revealed challenges, including a high prevalence of late-onset genotypes, variants of uncertain significance, and pseudodeficiency alleles that complicate decisions regarding treatment eligibility and timing [29]. The practical challenge is therefore not simply broader screening but the development of treatment-initiation criteria that integrate genotype, biomarkers of pathway function, and tissue imaging rather than relying on any single measure.

The encouraging early results of AAV9-LAMP2B therapy in Danon disease [26] sharpen rather than resolve the broader questions surrounding gene therapy. AAV vectors are now an established therapeutic platform, with several approved products and many others in development. Nevertheless, important challenges remain, including long-term durability of expression, strategies for redosing in the presence of pre-existing or treatment-induced a immunity; efficient delivery to the heart, skeletal muscle, and nervous system; and management of vector-related toxicities [27]. In lysosomal storage cardiomyopathies specifically, it remains unclear whether restoring the missing protein fully normalizes downstream autophagic flux or merely improves it. Answering this question once again depends on the development of reliable biomarkers of pathway function.

The most ambitious question is whether the convergent lesion itself can be treated directly. TFEB- and autophagy-directed therapies are conceptually attractive because they may be applicable across multiple disorders, yet they remain largely preclinical. Moreover, because autophagy is highly context-dependent, safe and titratable modulation will likely be required in place of broad or sustained activation [13,28]. Rigorous evaluation of these approaches will depend increasingly on improved human disease models, including patient-derived induced pluripotent stem cell (iPSC)-derived cardiomyocytes and organoids that more faithfully reproduce pathway dysfunction than many existing animal models [15]. Table 4 summarizes these priorities. Their common theme is that future progress may depend less on identifying additional substrates than on measuring and manipulating the shared cellular machinery that these substrates ultimately disrupt.

## 8. Conclusions

Fabry, Pompe, and Danon disease arise from different genetic defects and involve the accumulation of different substrates; however, they converge on a common failure: the cardiomyocyte can no longer adequately clear cellular material through the autophagy–lysosome system. The resulting mitochondrial dysfunction, hypertrophy, fibrosis, and electrical remodeling, therefore, represent variations of a shared pathogenic process. Viewing these disorders through a common mechanistic framework rather than as a collection of enzyme deficiencies helps explain why reducing substrate burden is beneficial but rarely curative, why intervention before fibrosis develops is so important, and why the most direct therapies either restore the missing protein at the entry point of the pathway or seek, more ambitiously, to repair the degradative machinery itself.

This perspective also highlights the field’s most important unmet need: a reliable means of measuring pathway function directly. For cardiologists and metabolic physicians alike, the practical message is to recognize the shared autophagy–lysosome–mitochondrial axis underlying otherwise unexplained hypertrophic, fibrosing, and arrhythmogenic heart disease to identify affected patients as early as possible and to match treatment to the level of the underlying lesion. In this sense, lysosomal storage cardiomyopathies are not merely a collection of rare disorders but a coherent—and increasingly treatable—group of diseases of cellular quality control.

## Figures and Tables

**Table 1:** Three core lysosomal/autophagic cardiomyopathies as variations on a shared defect.

Feature	Fabry Disease	Pompe Disease (GSD II)	Danon Disease IIb
**Gene/product**	GLA/α-galactosidase A	GAA/acid α-glucosidase	LAMP2/LAMP-2 (esp. LAMP-2B)
**Inheritance**	X-linked	Autosomal recessive	X-linked dominant
**Stored substrate**	Globotriaosylceramide (Gb3); lyso-Gb3	Lysosomal glycogen	Glycogen-laden autophagic vacuoles
**Core autophagy–lysosome lesion**	Glycosphingolipid storage with secondary impairment of autophagic flux [1,4]	Glycogen overload disrupts autophagic flux [6,7]	Primary failure of autophagosome–lysosome fusion [3,8,9]
**Cardiac phenotype**	Concentric LVH, diastolic dysfunction, fibrosis, and arrhythmia [1,2]	Infantile-onset severe hypertrophic cardiomyopathy [6,7]	Severe HCM, conduction disease/pre-excitation, and heart failure [3,9]
**Current and emerging therapies**	ERT; oral chaperone (migalastat); gene/substrate reduction in development [2,4]	ERT (alglucosidase/avalglucosidase alfa); AAV gene therapy, phase I/II [6,7]	Supportive care/transplant; AAV-LAMP2B gene therapy in trials [3,8]

Note. ERT, enzyme replacement therapy; GSD, glycogen storage disease; HCM, hypertrophic cardiomyopathy; LVH, left ventricular hypertrophy.

**Table 2:** Biomarkers in lysosomal storage cardiomyopathies, grouped according to what they report along the autophagy–lysosome–mitochondrial axis.

Biomarker/Class	What It Reflects	Main Role	Key Limitation
Plasma lyso-Gb3 (Fabry)	Glycosphingolipid storage	Diagnosis, screening, and individual monitoring	Insensitive in females and late-onset; no fibrosis signal
Urinary Glc4 (Pompe)	Glycogen-derived storage	Diagnosis and substrate burden	Weak link to myocardial status
Urinary GAGs (MPS)	Glycosaminoglycan storage	Diagnosis and substrate burden	Limited cardiac/valvular prognostic value
hs-cardiac troponin	Ongoing cardiomyocyte injury	Progression, prognosis	Nonspecific
NT-proBNP	Ventricular wall stress	Heart failure status and prognosis	Nonspecific
Inflammatory markers	Immune activation (early disease)	Emerging early-stage signal in Fabry	Not yet validated or specific
Native T1 mapping	Tissue composition (low T1 = sphingolipid storage)	Early detection and differential diagnosis	Pseudonormalizes as fibrosis develops.
LGE/ECV (CMR)	Replacement and interstitial fibrosis	Staging, prognosis	LGE misses diffuse fibrosis; needs gadolinium
Autophagic-flux/lysosomal-capacity marker (emerging: circulating LC3-II/p62, cell-free mtDNA, cardiomyocyte-derived EVs)	The failing machinery itself	Exploratory; none yet validated for cardiac use	The key unmet need

**Table 3:** Therapeutic strategies for lysosomal storage cardiomyopathies, arranged according to their point of action along the autophagy–lysosome pathway.

Strategy	Where It Acts/Mechanism	Examples (Disease)	Status	Key Limitation
Enzyme replacement	Replaces deficient enzyme; lysosomal uptake via M6P	Alglucosidase/avalglucosidase alfa (Pompe); agalsidase alfa/beta, pegunigalsidase alfa (Fabry)	Approved	Poor uptake into the heart/muscle; ineffective once fibrosis is established; lifelong infusions; antibodies
Pharmacological chaperone	Stabilizes amenable mutant enzyme; restores trafficking	Migalastat (Fabry, amenable variants)	Approved	Only amenable missense variants; in vivo amenability uncertain
Substrate reduction	Lowers the synthesis of the substrate	Glucosylceramide synthase inhibitors (Fabry)	Investigational	Acts upstream of the pathway lesion; cardiac efficacy unproven
Gene therapy	Restores the missing protein at source (AAV)	AAV9-LAMP2B/RP-A501 (Danon); AAV-GAA (Pompe); AAV-GLA (Fabry)	Phase 1 (Danon) and early phase (Pompe, Fabry)	Vector/immune toxicity; durability and flux normalization are unproven
Autophagy/TFEB-directed	Restores degradative capacity and flux directly	TFEB activation; mTORC1 modulation	Preclinical	Context-dependent; excess autophagy may harm

**Table 4:** Key open questions and research priorities for lysosomal storage cardiomyopathies.

Open Question	Why It Matters	Possible Approach
No circulating readout of autophagic/lysosomal flux	Therapy can be judged only by substrate or downstream surrogates, not by whether the pathway is working.	Develop and validate flux and lysosomal-capacity biomarkers; pair with cardiac magnetic resonance imaging
When to start treatment and in whom	Benefit depends on acting before irreversible fibrosis, yet newborn screening also flags late-onset, VUS, and pseudodeficiency cases.	Define treatment-initiation thresholds combining genotype, biomarker, and imaging
Durability and reach of gene therapy	Single-dose durability, anti-capsid immunity (redosing), and uneven delivery to the heart, muscle, and CNS remain unsettled.	Longer follow-up; capsid and promoter engineering; immune management
Does pathway-directed therapy translate?	Autophagy/TFEB modulation is the only strategy aimed at the convergent lesion but is unproven and context-dependent in vivo.	Cardiac-specific preclinical testing; safe, titratable TFEB modulation
Genotype–phenotype and sex differences	The same gene yields variable cardiac outcomes (notably in females with Fabry), shaping who needs early treatment.	Deep-phenotyping cohorts; modifier studies; better in vitro amenability assays
Better human models	Animal models imperfectly mirror human cardiac pathway failure.	Patient-derived iPSC-cardiomyocytes and organoids to test flux-level hypotheses

## Data Availability

No new data were created or analyzed in this study. Data sharing is not applicable to this article.

## Abbreviations

The following abbreviations are used in this manuscript:

AAV	adeno-associated virus
AMPK	AMP-activated protein kinase
BNIP3	BCL2/adenovirus E1B 19-kDa interacting protein 3
CLEAR	coordinated lysosomal expression and regulation
CMR	cardiac magnetic resonance
ECV	extracellular volume
ERT	enzyme replacement therapy
FUNDC1	FUN14 domain-containing protein 1
GAA	acid α-glucosidase
Gb3	Globotriaosylceramide
GLA	α-galactosidase A (gene)
Glc4	urinary glucose tetrasaccharide
HCM	hypertrophic cardiomyopathy
iPSC	induced pluripotent stem cells
LAMP-2	lysosome-associated membrane protein 2
LGE	late gadolinium enhancement
LSD	lysosomal storage disorder
LV	left ventricular
lyso-Gb3	Globotriaosylsphingosine
M6P	mannose-6-phosphate
MPS	Mucopolysaccharidosis
mTORC1	mechanistic target of rapamycin complex 1
NIX	NIP3-like protein X (BNIP3L)
NT-proBNP	N-terminal pro–B-type natriuretic peptide
PINK1	PTEN-induced putative kinase 1
PRKAG2	protein kinase AMP-activated noncatalytic subunit γ2
ROS	reactive oxygen species
SRT	substrate reduction therapy
TFE3	transcription factor E3
TFEB	transcription factor EB
TGF-β	transforming growth factor-β
VUS	variant of uncertain significance
